# Patient perspective on the climate health emergency

**DOI:** 10.1016/j.fhj.2025.100230

**Published:** 2025-03-31

**Authors:** Alice Joy

**Affiliations:** Royal College of Physicians, Belper, Derbyshire DE56 0QQ, England

**Keywords:** Climate, Patient

## Abstract

Patients and carers must be part of the move toward reducing waste, improving sustainability and tackling climate change in the current emergency. Without accurate, practical information they cannot contribute. Patients have fears associated with climate change and health, which are explored here.

This is not a scientific paper. For a person with limited access to journal articles, there is not an awful lot of validated information to be found and most of what is available appears to be opinion rather than data based. This is, rather, a reflection on what I have read and, perhaps more importantly, on what I have discussed with other patients or heard in passing.

It is no longer possible, even for the most uninterested individual, to ignore the crescendo of calls to action on climate change. Those who prefer to pretend that it doesn’t exist, or that there is no human cause of it, must still – and increasingly – fight their corner against undeniable evidence.

Patients and healthcare providers are caught in a vicious cycle in which the climate crisis is affecting human health on a global scale, while the very practice of curative care adds to the problem. There is work being done by clinicians to try to take, and encourage others to take, action to mitigate the effects of health care on the climate.^1^^,^^2^ There is also a movement toward discussing climate change with patients and educating us about what we can do ourselves, not only to reduce our effects on it, but also to protect ourselves in the face of it.^3^^,^^4^

There is, however, little evidence available (certainly to a patient) for co-production with patients and carers to understand and take direct action against causing further harm to the climate and the environment. As a lay person, if I look for published material on patients’ concerns with regard to the climate emergency, most of what I find has been written by psychiatrists about climate change-related anxiety.^6^ While this is undoubtedly an area of concern, I find it frustrating to see that so little has been done – or appears to have been done – with patients as partners. Indeed, the fact that such information is unavailable to me indicates that patients are generally excluded from the discussion. Even the government directs its advice on improving health and reducing emissions to professionals only.^5^ The point is, peer-reviewed papers are not often publicly available, so we often rely on information that may or may not be reliable.

This is a shame, because we lay patients (professionals are patients, too) are integral to addressing the problem. We absolutely do need educating about what we can do to help, but we also need to bring knowledge and ideas to the discussion and we need to be involved to be on board. Initiatives such as NHS Net Zero and Greener NHS need to be promoted and made readily available to patients. I have yet to see reference to these either in my local hospital or in my GP’s surgery, yet the Health Foundation’s survey of patients, which sought to determine patients’ attitudes to climate change and the NHS, found that 70% of respondents – and this was in 2021 – support the net zero ambition.^6^

There are many factors relating to the climate crisis that affect patients’ views on health and healthcare.

## Information and mis/disinformation

Without easily accessed, reliable information, patients can feel uncertain about the value of the information they do receive. Is climate change just a hoax? If there is a serious problem, why am I being bombarded with information that tells me I need yet another foot bath/raincoat/ornament at a knockdown price? Is the air I am breathing actually killing me? How much of what I read or hear can I actually believe? Where is the information that tells me what I can do about it?

## Climate change and health

According to the Health Foundation’s survey (ibid), almost half of respondents believed that climate change posed a real threat to human health at that time. It would be surprising if, in the intervening years, recognition of climate change as a threat to health had not increased. Furthermore, while a few years ago most of the threat seemed to be directed at developing nations, it is now understood by patients to have moved to our own shores. A quarter (25%) see climate change as one of the biggest threats to their health and it is seen as equally significant as accidents/injuries and mental health problems. (ibid) The fact that doctors and other health professionals are beginning to communicate with patients about what can be done both to reduce patient impact on climate and to improve health is welcome. Recommendations about walking or cycling, smoking cessation, reduction of meat in the diet and spending time in green spaces provide patients with practical actions they can take, which is good not only for their health but also gives hope in what can feel like a hopeless situation.

## Future generations

A serious concern, not just for patients but for everyone, is the impact that climate change has and will continue to have on future generations. Not all patients are aware of the threat to food production, but those who are, and who are aware of the potentially catastrophic result of soil damage and depletion, worry about how their children, grandchildren and generations to come will eat. They worry, too, about mass migration resulting from areas of the world becoming uninhabitable. All of this either has an immediate impact on health – mental and physical – or may have in months and years to come.

## Inequalities

Patients in poverty are particularly vulnerable, as we know, and have much greater difficulties accessing not just healthcare, but also healthy diets, green spaces and clean environments. Climate change is not going to improve the situation. People who are less well off also face difficult decisions about how to use the resources they have; whether to be warm or fed. Those who are comfortable enough to heat their homes, if they are climate aware might feel guilt at contributing to the problem yet risk illness if they are cold.

## Lack of control

All of this adds up to lack of control, which is known to generate anxiety. I have no doubt that mental health affects physical health, but as a lay person I am unable to access evidence of that. Searching the internet brings up NHS information on the way that physical health impacts on mental health and the only reference I can find for the other way round is on Healthline, which will not admit me without my agreement to use of cookies.

This is a problem. Patients lack control, which breeds anxiety, which, I believe (and so do many other patients), can be manifested in physical ways. Our access to information is somewhat limited and, where it isn’t, can be unfathomable, misleading or downright wrong. Information needs to be accessible both in format and in comprehensibility. The value of the movement to help doctors and other healthcare professionals discuss climate change with patients; to educate and inform with regard to lifestyle and what we, as individuals, can do, is immense and to be wholeheartedly welcomed. However, it needs to be done with us, not to us. It is understood that not all patients will be receptive, that time constraints are a major concern and that it will not always be an easy conversation, but we are in an emergency. If people (and that includes all of us) are to understand why action is vital, they need to understand the meaning of the word.

## Living in constant fear

There is, of course, the danger of the human reaction to any fearsome thing that we encounter; if it doesn’t kill us immediately, we begin to get used to the idea and consider it to be less important than we did. That is why constant vigilance, information and education are vital. The elephant in the room is the body of policy makers at the top of the tree, who need to improve public health, public transport and public awareness.

Another real fear for patients is that climate change will render healthcare provision unsustainable. The media seem to be very fond of criticising the NHS – sometimes with good reason – but rarely praise it or support it, so patients may have a heavily biased view of how bad things are. However, we know that waste is a major problem, for example with billions of disposables and with medical gases. We know, too, from NHS workers, that overwork and burnout are a reality. We have seen the ‘brain drain’ as British-trained doctors opt to go abroad to work and we hear GPs pleading for understanding. So, is wanting good care selfish? Probably, but even we oldies who are societally disposable have families and friends who value us. And, importantly I think, we want to help, not just add to the difficulties.^7^

## Conclusion

If we are to act on climate change, we must do it as a single body. Doctors and patients together have a stronger voice than either group alone. Those who are not yet patients must join the discussions, too. I know that there are many people ‘out there’ who are beavering away at finding better, more sustainable ways of doing things, but what I hear is a mewing, not a shouting. Those who shout are in danger of incurring penalties, so we must all shout together, and louder.^8^ Maintaining good health is more sustainable than treating bad health, so the time and funding required to promote and support public health would be a great investment for the future. Let us try to keep the number of patients to a minimum.

## Funding

This research did not receive any specific grant from funding agencies in the public, commercial or not-for-profit sectors.

## CRediT authorship contribution statement

**Alice Joy:** Conceptualization, Writing – review & editing.

## Declaration of competing interest

The authors declare that they have no known competing financial interests or personal relationships that could have appeared to influence the work reported in this paper.
